# Maximal Oxygen Uptake, VO_2_ Max, Testing Effect on Blood Glucose Level in Adolescents with Type 1 Diabetes Mellitus

**DOI:** 10.3390/ijerph19095543

**Published:** 2022-05-03

**Authors:** Kristi M. King, Timothy McKay, Bradly J. Thrasher, Kupper A. Wintergerst

**Affiliations:** 1 Department of Health and Sport Sciences, University of Louisville, Louisville, KY 40292, USA; 2 Norton Children’s Hospital, Louisville, KY 40202, USA; timothy.mckay@nortonhealthcare.org (T.M.); bradly.thrasher@louisville.edu (B.J.T.); kupper.wintergerst@louisville.edu (K.A.W.); 3 Wendy Novak Diabetes Center, Pediatric Endocrinology, School of Medicine, University of Louisville, Louisville, KY 40202, USA

**Keywords:** maximal oxygen uptake, VO_2_ max, blood glucose, type 1 diabetes mellitus (T1DM), adolescents, exercise testing, pediatric, clinical exercise

## Abstract

Assessing maximal oxygen uptake (VO_2_ max) is generally considered safe when performed properly for most adolescents; however, for adolescents with type 1 diabetes mellitus (T1DM), monitoring glucose levels before and after exercise is critical to maintaining euglycemic ranges. Limited guidance exists for glucose level recommendations for the pediatric population; therefore, the purpose of this retrospective clinical chart review study was to determine the effects of VO_2_ max testing on blood glucose levels for adolescents with T1DM. A total of 22 adolescents (mean age = 15.6 ± 1.8 years; male = 13, 59.1%) with a diagnosis of T1DM participated in a Bruce protocol for VO_2_ max from January 2019 through February 2020. A statistically significant reduction in glucose levels between pretest (<30 min, mean = 191.1 mg/dL ± 61.2) and post-test VO_2_ max (<5 min, mean = 166.7 mg/dL ± 57.9); *t*(21) = 2.3, *p* < 0.05) was detected. The results from this current study can help guide health and fitness professionals in formulating glycemic management strategies in preparatory activities prior to exercise testing and during exercise testing.

## 1. Introduction

One of the tenets of the sports medicine field is to advance and integrate scientific research to provide educational and practical applications of exercise science and sports medicine. For individuals engaging in physical activity at any level, whether it is recreational physical activity or competitive sports, there is clear, scientifically based guidance regarding exercise testing and prescription for health and fitness professionals to employ with healthy individuals as well as those living with chronic illnesses [1]. One component of health-related physical fitness is cardiorespiratory fitness (CRF), the body’s ability to perform large-muscle, dynamic, moderate-to-vigorous-intensity exercise for prolonged periods of time. Assessing the maximal oxygen uptake (VO_2_ max) the body is able to use during exercise is an established exercise test for determining CRF and is more predictive of long-term survival than is any traditional risk factor or other measured physiologic parameter [2]. VO_2_ max testing provides a measurement of the relative amount of oxygen consumption per an amount of work. For example, an improved VO_2_ may allow one to run longer at the same speed or faster with the same relative effort [3].

The graded exercise test used to elicit VO_2_ max is aggressive in nature to achieve a maximal response from the participant. Under stress conditions, the hypothalamus controls many hormone secretions to adjust glucose metabolism and energy production. Glucose secretion and uptake are under the control of nervous and hormonal factors such as catecholamines, cortisol, glucagon, growth hormone, and insulin, and all have an immediate impact [4]. Even though exercise testing is generally considered safe when performed properly for most individuals, maximal- or vigorous-intensity exercise testing does pose some risk [5,6,7,8]. Specifically, for individuals with type 1 diabetes mellitus (T1DM), the risk of hyperglycemia in the initial portion of exercise testing and the risk for hypoglycemia following completion of testing both present themselves. Monitoring glucose levels before and after physical activity is fundamental to maintaining glucose levels in euglycemic ranges during and after exercise [9].

Unfortunately, understanding of safety parameters and the effect of CRF testing on adolescent populations is limited and in need of research [10]. Glucose level recommendations have yet to be established for adolescents diagnosed with T1DM who participate in VO_2_ max exercise testing. Although a decrease in glucose may be expected throughout and immediately after exercise testing, a minimal pretest glucose setpoint has not been established to reduce the risk of hypoglycemia. Therefore, the purpose of this study was to examine the impact of VO_2_ max testing on blood glucose levels for adolescents with T1DM.

## 2. Materials and Methods

### 2.1. Study Design and Setting

This cross-sectional, non-interventional, retrospective chart review study was conducted at a nationally certified pediatric diabetes care and academic medical center located in the Southeast region of the United States. At this center, pediatric endocrinologists, registered nurses, registered dieticians, certified diabetes educators, and clinical exercise physiologists treat pediatric patients diagnosed with T1DM up to 26 years of age. The study was approved by the University Institutional Review board. Retrospective clinical chart reviews were conducted of clinical pediatric sports medicine and physical activity program patients with a diagnosis of T1DM who participated in a Bruce protocol for VO_2_ max from January 2019 through February 2020.

### 2.2. Participant Characteristics

The baseline characteristics of study participants are displayed in Table 1.

### 2.3. Measures

Socio-demographic, anthropometric, diabetes monitoring and treatment plans, and hemoglobin A1c (HbA1c) levels data were retrieved from patients’ medical records, maintained in the clinical database, as part of standard practice at each patient’s appointment in the diabetes clinic. The socio-demographic and anthropometric characteristics utilized were participant’s age, date and duration of T1DM diagnosis, ethnicity, race, gender, insurance type, and body mass index (BMI) percentile. Diabetes monitoring was assessed (including whether the participant used a continuous glucose monitor (CGM)), and the type of treatment plan was recorded. The hemoglobin A1c (HbA1c) level was obtained from that day’s clinical lab measures at check-in. HbA1c, the most prevalent and accessible measure in determining glucose control, was used as an indicator of the average blood glucose levels over the past 3 months. Adolescents managing T1DM should strive for HbA1c levels less than 7%, as an elevated HbA1c level is known to increase the risk for diabetes-related complications [11]. Blood glucose levels and VO_2_ max data were obtained from sports medicine records collected by clinical exercise physiologists in the sports medicine clinic.

### 2.4. Preparatory Activities Prior to Exercise Testing

Upon registration for an exercise testing appointment, participants were instructed to not eat a heavy meal two hours prior to testing, to maintain their insulin regimen as they would on a regular day, and to dress in exercise attire (e.g., shorts, t-shirt, athletic shoes). Upon arrival to the sports medicine clinic, participants’ blood glucose value was screened by a clinical exercise physiologist. If the blood glucose was >250 mg/dL the clinical exercise physiologist obtained urinary ketones with the next void. If urinary ketones were moderate or large, the participant was excluded from participating in VO_2_ max testing at that time. If the blood glucose was >300 g/dL and the participant had no or small ketones, the clinical exercise physiologist instructed the participant to give a conservative insulin correction of 50% their calculated correction dose.

Approximately 30 min prior to VO_2_ max testing, a pretest blood glucose sample was taken using a point-of-care glucometer and lancet device. Upon determination that blood glucose levels were in the safe range for physical activity, the clinical exercise physiologist conducted the VO_2_ max test. Upon completion of the VO_2_ max test, a post-test glucose level check was conducted within 5 min using the same glucometer for all participants.

### 2.5. Exercise Testing Procedures

A Bruce protocol [12] for VO_2_ max tests, a valid and reliable measure for assessing cardiorespiratory fitness, was performed on a Woodway ELG treadmill while the participants were connected to the Parvo Medics metabolic gas exchange analyzer by way of respiratory mask. Participants walked on a treadmill in 3 min bouts, starting at 1.7 mph (45.6 m·min^−1^) and 10% grade. At each stage, the speed was increased by either 0.8 or 0.9 mph (21.4 or 24.1 m·min^−1^) and the grade was increased by 2%. This test lasted approximately 10–20 min.

If following the graded exercise test the participant’s blood glucose was found to be <70 mg/dL on the glucometer, the participant was treated for hypoglycemia with 15 g of rapid-acting carbohydrate. Blood glucose was then rechecked at 15 min. This process was repeated until their blood glucose was >70 mg/dL. Blood glucose and VO_2_ max data stored within REDCap on a secure server in the sports medicine program data were linked to the clinical database by the researchers utilizing the patients’ medical record numbers. All clinical data were retrieved from that same-day appointment for each participant. Once data were collected and merged, the full dataset was de-identified for analysis.

### 2.6. Data Analysis

All statistical analyses were conducted using IBM SPSS 27.0 (IBM Corp., Armonk, NY, USA). Descriptive statistics and frequencies for socio-demographic, anthropometric, diabetes monitoring and treatment plans, HbA1c levels, and pre- (<30 min) and post-test (<5 min) blood glucose levels were calculated. Shapiro–Wilk’s test (*p* < 0.05) [13,14], histograms, Norman Q–Q plots, and box plots were employed to test the normality of the distribution of the data. A paired-samples *t*-test was employed to detect differences in blood glucose levels from pretest to post-test. *p*-Values of <0.05 were considered as statistically significant.

## 3. Results

Retrospective VO_2_ max data were analyzed from a total of 22 adolescents (*N* = 22; mean age = 15.6 ± 1.8 years; male = 13, 59.1%) (see Table 1). Most of the participants identified as non-Hispanic (*n* = 20, 90.9%), and over three-quarters identified as White (*n* = 17, 77.3%). Continuous glucose monitors were worn by 13 of the 22 participants (59.1%). Their average HbA1c prior to participating in the VO_2_ max test was 8.9% ± 1.8. The average BMI, based on age and sex, was in the 67th percentile ± 17.7. The average VO_2_ max peak was 43.4 mL/kg/min ± 6.4 (See Table 2).

Pre- and post-test blood glucose measurements were obtained from 22 participants. The results of a Shapiro–Wilk’s test indicated that the pre- and post-glucose data were normally distributed, and a visual inspection of their histograms, Norman Q–Q plots, and box plots showed that the glucose scores were normally distributed at pretest with a skewness of 0.107 (SE = 0.49) and a kurtosis of −0.868 (SE = 0.95) and at post-test with a skewness of 0.657 (SE = 0.49) and a kurtosis of −0.015 (SE = 0.95). A paired-samples *t*-test was employed to detect a statistically significant reduction in glucose levels between pretest (<30 min, mean = 191.1 mg/dL ± 61.2) and post-test VO_2_ max (<5 min, mean = 166.7 mg/dL ± 57.9); t(21) = 2.3, *p* < 0.05).

## 4. Discussion

It is well established that significant changes in blood glucose concentration during physical activity can lead to hypoglycemia or hyperglycemia and, if not prevented or treated quickly and properly, can lead to a medical emergency [1,9,15,16,17,18,19,20,21,22,23,24,25,26,27]. This current study sought to examine if there was a significant drop in blood glucose levels after VO_2_ max testing, yet it is unique in that it specialized in a pediatric population of adolescents. Results from a recent retrospective study with adults with T1DM (mean age = 32 years, SD ± 13; range 18–65 years) who participated in VO_2_ max exercise testing using a cycle ergometer did not demonstrate statistically significant glucose levels from pretest to post-test [28], which aligned with similar results from other studies [29,30]. The conflicting results from the present study may be attributed to differences in the age of participants (and in body composition and hormones) and possibly the modality used during testing.

Given that individuals with T1DM are recommended to participate in daily moderate-to-vigorous-intensity physical activity [31], and general guidance for glucose targets as well as nutritional and insulin dose adjustments to protect against exercise-related glucose excursions are available [9,20,21,26,27], fear of activity-related hypoglycemia has been regularly cited as a barrier to physical activity [32,33]. Health care providers wishing to prescribe even modest increases in intensity levels of daily activity, such as walking and/or jogging, or sport participation that may include moderate-to-vigorous-intensity activity to their patients with T1DM may consider VO_2_ max testing as a first step in establishing safety precautions and working toward the adoption and maintenance of an active lifestyle. For example, participation in sports is touted as a beneficial means for adolescents to accumulate physical activity [34,35]. However, caution must be taken if prescribing sport only without the engagement in additional physical activity. This is because many adolescents who participate in a single sport often do not meet sufficient physical activity recommendations. A recent study involving 153 children and adolescents diagnosed with T1DM demonstrated this fact [36]. Although almost two-thirds of the participants reported playing one or more sports in the previous year, they were only physically active for at least one hour or more on an average 3.5 days per week, with less than 8% of the children and adolescents in the study meeting the recommended duration of one hour and frequency of seven days per week of physical activity.

The results from this current study may help guide health and fitness professionals in formulating glycemic management strategies in preparatory activities prior to exercise testing and during exercise testing. A pre-exercise glucose level of 90–250 mg/dL is suggested in order to prevent symptoms of hypoglycemia and to minimize hyperglycemia [9,11,26]. Considering that the adolescents in this current study experienced a 24.4 mg/dL drop in glucose levels from pretest to post-test, the implications of these results have clinical and practical importance. These results can and should be used to help inform patients and practitioners in clinical care decision making and the formulation of glycemic management strategies. Similar research findings suggest that patients and clinical care teams understand the glycemic changes that occur during progressive exercise so that nutritional and medicinal preparatory routines are safely established [28]. To ensure safe exercise performance ahead of exercise testing, practitioners, physiologists, and patients should be aware of the interindividual responses to VO_2_ max testing and treat each case accordingly. With the assistance of a clinical exercise physiologist, physicians can incorporate individualized recommendations for increasing physical activity and/or exercise prescriptions into their clinical practices [37]. Physicians and medical care teams can prescribe physical activity and sport participation when designing treatment plans and refer their patients to qualified health and fitness professionals such as athletic trainers, strength and conditioning coaches, and physical educators who coach or train adolescent athletes diagnosed with T1DM. These protective measures that are grounded in scientific evidence [9,11,38] suggest that adolescent patients diagnosed with T1DM can complete maximal exercise testing without fear of inducing hypoglycemia if the necessary safety precautions as described in this study are taken.

### Limitations and Future Research

Various limitations have been identified in this study. Although all participants were instructed to avoid eating greater than 60 g of carbohydrates prior to exercising, unless hypoglycemic, the study was not controlled for nutrition. Future studies should analyze dietary practices leading into exercise testing. In addition, participants were also instructed to administer insulin per their routine standard of care to create a “real-world” testing situation for this study. Future studies could benefit from more restrictive insulin use parameters.

The data in this study were derived from a retrospective chart review of clinical patients who participated in a pre–post VO_2_ max test at a newly established (2018) clinic serving only pediatric patients diagnosed with T1DM up to 26 years of age from January 2019 until February 2020. In March 2020, non-emergency clinical operations were suspended due to COVID-19 safety precautions protocol, and sports medicine programming and study endeavors resumed in August 2021, which further limited our total sample size. At the time of the study, there were no matched control group data available. Next, although the clinic houses the only pediatric endocrinology sports medicine program in the state, the homogeneity of the participants in race and ethnicity does not make the findings generalizable to adolescents in other areas. Future research will benefit from a more extensive and longitudinal review of the pre- and post-VO_2_ max testing windows to further understand what variables influence blood glucose variability

## 5. Conclusions

The results from this retrospective VO_2_ max testing study on blood glucose levels in adolescents with T1DM can add to the scientific literature for sports medicine programs that provide clinical care to individuals and their families through patient-centered and community education as well as clinical research. Regardless of sport or physical activity, care is focused on improving the health, safety, and athletic performance of every child and young adult with T1DM. Knowing that a significant drop in glucose levels during VO_2_ max testing may occur with their adolescent patients, health and fitness professionals can discuss and implement preventive glycemic management strategies prior to exercise testing and during exercise testing.

## Figures and Tables

**Table 1:** Characteristics of the Participants, *N* = 22.

Characteristics	Mean ± SD or *n*, %	Range (Minimum–Maximum)
Age, years	15.6 ± 1.8	13–20
Duration of T1DM diagnosis, years	7.1 ± 4.9	>1–16
Height, centimeters	170.9 ± 8.3	157.3–187.4
Weight, kilograms	67.1 ± 13.4	44.6–107.3
BMI percentile, *n* = 21	67th percentile ± 17.7	26–99
HbA1c level, *n* = 21	8.9 ± 1.8	6.1–14.9
Gender	Male, *n* = 13, 59.1% Female, *n* = 9, 40.9%	-
Ethnicity	Non-Hispanic, *n* = 20, 90.1%	-
Race	White, *n* = 17, 77.3% Black or African American, *n* = 3, 13.6% Unknown or Not Reported, *n* = 2, 9.1%	-
Treatment Plan	CGM only, *n* = 2, 9.1% Insulin pump only, *n* = 4, 18.2% Insulin pump integrated with CGM, *n* = 9, 40.9% MDI, *n* = 7, 31.8%	

Note. Data are presented as mean ± standard deviation (SD) or number of participants (*n*), percent (%); BMI, body mass index percentile; HbA1c, hemoglobin A1c; CGM, continuous glucose monitor; MDI, multiple daily injections.

**Table 2:** VO_2_ max testing measurements, *N* = 22.

Characteristics	Mean ± SD	Range (Minimum–Maximum)
VO_2_ max, mL/kg/min, *n* = 21	43.4 ± 6.4	29.3–50.5
Peak HR bpm, *n* = 19	192.3 ± 21.3	119–212
Glucose, mg/dL pretest, *n* = 22	191.1 ± 61.1	96–296
Glucose, mg/dL post-test, *n* = 22	166.7 ± 57.9	83–297

Note. Data are presented as mean ± standard deviation (SD); Peak HR bpm, heart rate beats per minute.
